# Mechanical Properties of the AlCu4Mg1 Alloy Joint Manufactured by Underwater Friction Stir Welding

**DOI:** 10.3390/ma17081722

**Published:** 2024-04-09

**Authors:** Ireneusz Szachogłuchowicz, Lucjan Śnieżek, Adam Wójcik

**Affiliations:** Faculty of Mechanical Engineering, Military University of Technology, 2 gen. S. Kaliskiego St., 00-908 Warsaw, Poland; lucjan.sniezek@wat.edu.pl (L.Ś.); adam.wojcik@student.wat.edu.pl (A.W.)

**Keywords:** FSW, UWFSW, aluminum alloy, mechanical properties, microstructure, AlCu4Mg1

## Abstract

The manuscript presents the results of butt joining of 3-millimeter-thick AlCu4Mg1 alloy sheets using the FSW (friction stir welding) and UWFSW (underwater friction stir welding) methods. The aim of the research is to verify the influence of the water environment on the FSW friction welding process. The article checked three sets of joint parameters. The parameters differed in tool rotation speed and feed rate. The same sets of parameters were used for the FSW and UWFSW fusion techniques. With the supplied devices, metallographic sections are cross-sectioned, and the power supplies are subjected to a light microscope. Microhardness tests and the influence of the heat-affected zone were carried out. A monotonic test was performed. A monotonic test is available, extended with a visual correlation test. The obtained cracked fracture surfaces were examined using a scanning microscope. An analysis of the microfractographic cracking process was carried out. The obtained results did not show any improvement in the strength properties of the obtained joints made using the UWFSW technique when using a scanning microscope.

## 1. Introduction

The aluminum alloys from the 2XXX series are famous for their unique properties, including strength properties, increased ballistic resistance, and resistance to stress corrosion cracking [1,2]. These characteristics make it a highly suitable choice for applications in aircraft and vehicles [3]. Welding precipitation-hardened aluminum alloys, for example, AlCu4Mg1, presents certain challenges, including susceptibility to hot cracking, significant weakening of strengthening phases, and the occurrence of porosity. To overcome these difficulties, nonconventional welding techniques have been explored. These alternative methods make it possible to create complex structures while reducing the tensile strength of the joint [4,5,6,7]. By implementing these innovative welding techniques, it becomes possible to achieve high-quality welds and preserve the integrity and strength of the alloy, thus enabling the fabrication of complex components for various applications in the aerospace and automotive industries. Finding suitable welding techniques and filler materials that ensure compatibility and maintain the integrity of the joint is essential.

**Figure 1:**
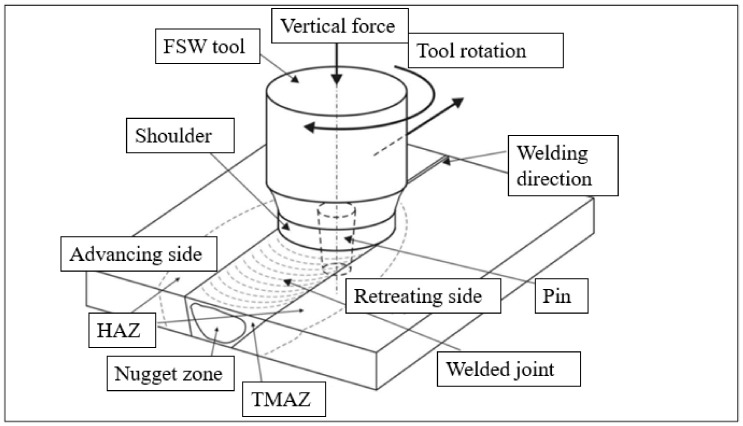
The process of joining using the FSW method.

One of the emerging joining techniques is the FSW method. This method combines metals and their alloys in a plasticized state. This technique is particularly effective in joining materials considered difficult to weld using traditional welding techniques. The FSW friction welding process involves the use of a rotating, cylindrical tool that contacts the materials to be joined while simultaneously advancing the rotary tool along the joint seam, as shown in Figure 1.

The tool is then moved along the seam of the joint. Heat is generated through friction produced by the tool, causing the material to soften and form a solid joint with a combination of mechanical and plastic deformation. This process allows the materials to mix without reaching their melting point, and the rotation of the tool facilitates mixing in the joint area. Once the joining is complete, the tool is removed from the work zone [8,9]. The ability to joint aluminum alloy parts using FSW has significant advantages in the production of aerospace structures. It reduces cost and weight while maintaining comparable or even better strength properties compared to traditional joining methods that use additional joining elements. FSW provides a reliable and efficient way to create strong joints in aluminum structures, making it a valuable technique in the aerospace industry. Nevertheless, one of the drawbacks of conventional friction stir welding (FSW) techniques used for heat-treatable aluminum alloys is the significantly lower tensile strength of the joint compared to the native material [10].

Recently, greater emphasis has been placed on improving the strength properties of FSW joints by controlling the temperature in the joint zone. A common solution is to immerse the rotary tool and the joined element in water [11,12,13]. The analysis of the results of joints made using the UW-FSW method showed that cooling had an impact on the quality of the joints. The increased speed of heat dissipation resulted in a reduction in the number of defects and imperfections in the joint zone. The results from the monotonic tensile strength tests indicate a significant improvement in joint strength thanks to the use of UW-FSW [14]. Joints using the UW-FSW technique were made on an AA2017 aluminum alloy. The attempts made concerned welding 5-millimeter-thick sheets. In parallel with experimental research, model research was carried out to analytically describe the physical phenomena occurring during joining. The results obtained showed a strong correlation [15]. The current study investigated the joining of AA5052 aluminum alloy sheets using both traditional friction stir welding and underwater friction stir welding techniques. The joined elements were 1 mm in thickness. In the case of the UW-FSW connection, the tool and the welding plates were completely immersed under water. The underwater welding process aims to improve the strength and mechanical properties of aluminum alloys by reducing heat dissipation. Comparative analysis shows that UW-FSW welding, due to its lower heat emission compared to conventional friction stir welding, provides excellent mechanical properties, deformability, and microstructure. Therefore, these findings suggest its usefulness in the automotive industry [16].

Researchers, such as Fratini, have observed improved joint properties when using this immersed procedure compared to standard joints [17]. Additionally, Sakurada has pioneered the use of aluminum alloys submerged in rotary friction stir welding, demonstrating that sufficient friction for welding can still be generated even with the submerging of the welded plates [18]. Their research focuses on generating refined grain structures in materials, resulting in improved mechanical properties. By effectively controlling the temperature through submerged techniques in FSW, researchers have achieved significant advancements in weld properties. This breakthrough has the potential to enable the production of high-quality joints in aluminum alloys. These findings have profound implications for the manufacturing industry, as they open up opportunities to develop stronger and more durable components through enhanced welding processes. The UWFSW technique (underwater friction stir welding) is based on the use of external liquid cooling, which fills the tank and covers the layer of material to be welded. The results of the research presented in the article [19] indicate that making a connection in an aqueous environment should have positive effects in the form of increasing the strength of the joint.

In summary, in the UWFSW technique, water surrounding the mixing zone provides a number of advantages, among them: it reduces the amount of heat, increases the rate of cooling of the material, which helps in the formation of fine microstructures in the welding zone, and improves mechanical properties [20]. Currently, the UWFSW technique is becoming an increasingly promising direction of development in the field of friction joining.

This paper aims to study the effects of tool speed (750–1500 rpm) and welding speed (100–200 mm/min) on the basic properties of AlCu4Mg1 friction stir-welded joints. We compared how the use of external walking in the form of water—UWFSW with unchanged parameters—affects the strength test values, microstructure, and microhardness distribution.

## 2. Experimental Procedures

The AlCu4Mg1 alloy is the tested material, one of the most durable alloys from the 2XXX series, whose main components are copper and magnesium. The AlCu4Mg1 alloy was heat treated—saturated, cold-deformed, and then naturally aged. The heat treatment processes carried out in this way resulted in very good strength properties. The basic chemical composition of the alloy, according to the manufacturer’s data, is presented in Table 1.

Table 2 presents the basic strength properties of the AlCu4Mg1 aluminum alloy obtained during a static tensile test.

The material used for testing the AlCu4Mg1 alloy is considered difficult to weld or unweldable using conventional methods. Aluminum alloys of the 2XXX series are more sensitive to hot cracking in the melted zone and partially melted zone and to a decrease in mechanical properties in the heat-affected zone. A joining temperature that is too high causes problems with cracking and porosity.

### Experimental Setup

**Figure 2:**
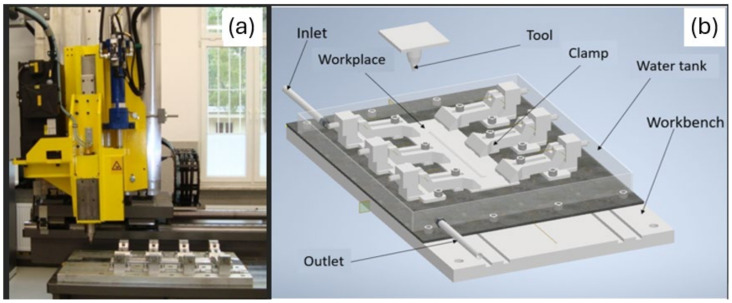
(**a**) FSW ESAB LEGIO 4UT welding device. (**b**) Diagram of the container for carrying out the joining process using the UWFSW technique.

The research methodology includes carrying out the friction stir welding (FSW) and underwater friction stir welding (UWFSW) processes, in each case using the ESAB FSW Legio 4UT machine (ESAB, Warsaw, Poland) shown in Figure 2a. The elements were joined together during one straight pass of the machine tool in a given direction along the joined elements, obtaining a continuous seam along the entire length of the joint. The connection was made on sheets of steel with dimensions of 1000 × 2000 mm and a thickness of 3 mm produced by the AMAG rolling GmbH plant in Austria. The connection parameters were selected based on literature data. The main assumption of the selected parameters was to check and compare how changing the tool feed speed v while modifying the rotational speed ω affects the strength properties of the FSW and UWFSW joints of the tested AlCu4Mg1 aluminum alloy. The selection of parameters included changes in the rotational speed of 750–1500 rpm and the welding speed of 100–200 mm/min, which, together with the adopted sample designations, were summarized in the table (Table 3). The results of the research presented in the article [20] indicate that making a connection in an aqueous environment should have positive effects in the form of increasing the strength of the joint. The water temperature before the connection was made was 293 Kelvin. A constant axial force of 17 kN and an inclination angle of 2° for the ESAB 2902 were set. Additionally, the heat quantity coefficient for the applied bonding parameters was calculated from Equation (1), which is also included in Table 3.

Q = ω/v(1)

where:

Q—quantity of heat (J/mm);
ω—tool rotation speed (rpm);
v—tool feed speed (mm/min);
W—determination of samples combined using the UWFSW method.

**Table 3:** The joining parameters used for each sample, along with the marking.

Sample Designation	Tool Rotational Speed ω (rpm)	Tool Feed Speed v (mm/min)	Quantity of Heat Q (J/mm)
I	750	200	3.75
II	1040	143	7.27
III	1500	100	15
I_W	750	200	3.75
II_W	1040	143	7.27
III_W	1500	100	15

A container was designed to carry out the UWFSW process of the AlCu4Mg1 aluminum alloy (Figure 2). The sheets were attached to the container using clamps. Then, the container was filled with water to a height of approximately 7 mm so that the joined material was completely covered with a layer of liquid, and the working tool and the pin were also surrounded by water during the test of joining the elements. The container is equipped with two pipes supplying liquid to the tank and draining it, connected to the radiator to ensure a constant temperature of the liquid in the tank close to the initial temperature.

The tool used to connect the elements is ESAB2902, with the following dimensions and parameters, included in Table 4.

**Figure 3:**
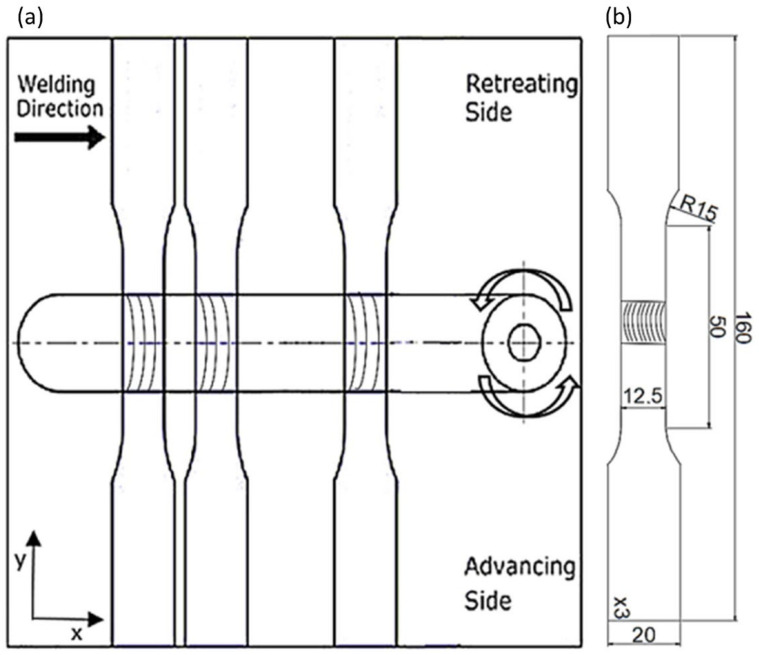
Diagram of the process of joining samples with a given geometry (**a**) and the geometry of the sample for a static tensile test (**b**).

After the joining process was carried out using the FSW and then UWFSW methods, samples were cut from the obtained joint section for analysis of strength parameters. The geometry of the sample was cut from the sheet metal perpendicular to the axial direction of the obtained joint in accordance with the ISO 6892-1 standard [21] and is shown in Figure 3.

Samples for static tensile testing were made in accordance with the ASTM E8/E8M–13a standard [22]. The research used the Instron 8802MTL universal testing machine (INSTRON, Warsaw, Poland) with the company’s WaveMatrix computer software (Instron, Norwood, MA, USA). An extensometer (2620-604, INSTRON, Warsaw, Poland) with a measurement base of 50 mm was used to measure the strain. During the test, the system recorded load and deformation values. Based on DIC (digital image correlation) using the DIC Dantec Q-400 system, sample properties were tested by analyzing the sample surface. The tests were performed for 3–5 samples for each joining parameter. Representative samples of particular types of parameters were selected for a detailed analysis. Conducting microscopic examinations was possible thanks to the appropriate preparation of samples using metallographic preparation devices. As part of the preparation, the samples were cut in the axial direction using a diamond saw, then embedded in resin—hot insertion, polished with sandpaper of the following gradations: 320, 500, 800, 1200, and 2000, and finally polished with diamond pastes. The samples were etched with Kroll’s reagent (consisting of 20 mL of water, 5 mL of nitric acid, 1 drop of hydrofluoric acid, and 2 drops of hydrochloric acid), and the etching time was 15 s. Microscopic examinations aimed at examining the macrostructure of the obtained sample joints were carried out on a test stand with an OLYMPUS LEXT OLS 4100 confocal laser microscope (Olympus IMS, Waltham, MA, USA). Microhardness was also measured using the Vickers method on a Struers DURA SCAN 70 microhardness tester (Struers, Copenhagen, Denmark) in accordance with the ASTM E384 standard [23]. As part of the tests, three series of impressions were carried out at three different distances in each line, 74 points from the upper surface of the sample, respectively: 0.5 mm, 1.5 mm, and 2.5 mm, applying a load of 0.98 [N]. Observations of the fracture surface microstructure were made using the Jeol JSM-6610 scanning electron microscope (JEOL, Tokyo, Japan) on the obtained fractures of samples subjected to static tensile testing. Selected micro-sections of fracture surfaces were analyzed for both samples after the FSW and UWFSW processes.

## 3. Results

**Figure 4:**
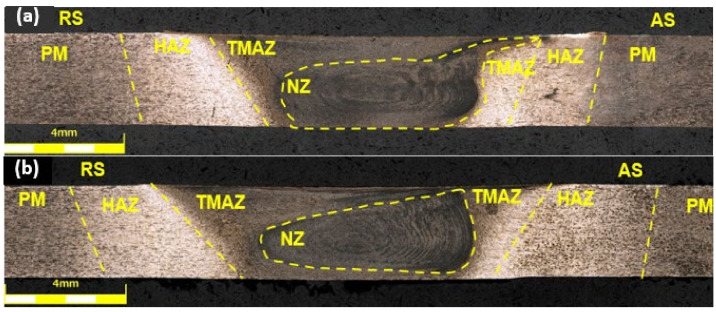

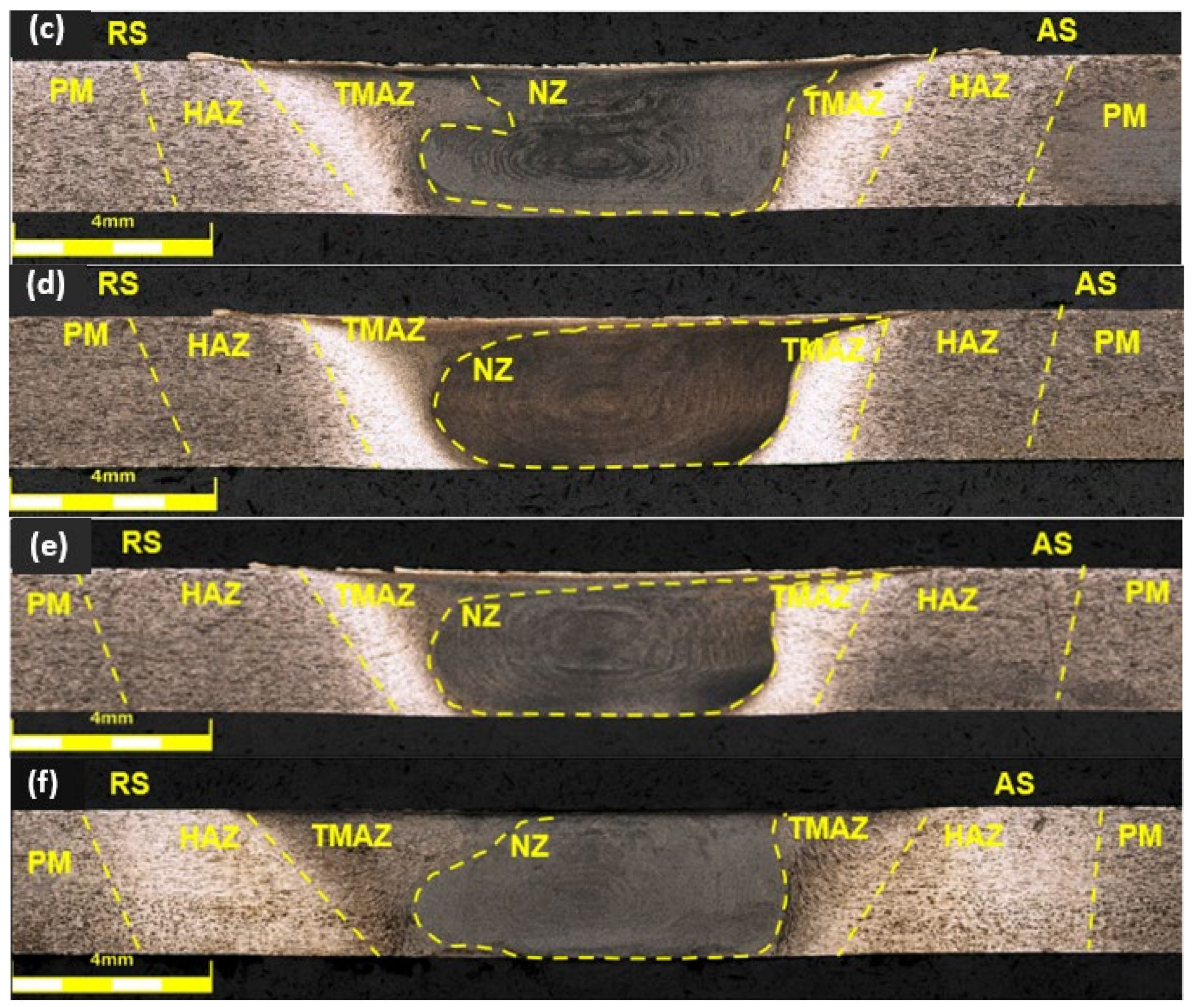
Optical microscopy images of the FSW and UWFSW junction: (**a**) I, (**b**) I_W, (**c**) II, (**d**) II_W, (**e**) III, and (**f**) III_W.

Using the selected operating parameters of the tool, all connections for both FSW and UWFSW were successfully completed. The obtained connectors were analyzed visually and aesthetically, and no defects were noticed on their surface. A difference in the appearance of the connectors was observed. The amount of flash increased with the tool rotation speed. A broader analysis of the connection quality was performed using light microscopy. The macrostructures of the cross-sections of the obtained joints are shown in Figure 4, and the welding parameters for each micrograph were: (a), (b)—750 rpm and 200 mm/min; (c), (d)—1040 rpm and 143 mm/min; and (e), (f)—1500 rpm and 100 mm/min.

In each of the sample images, characteristic joint zones differing in microstructural structure were distinguished, and the following designations were used to describe them: NZ—nugget zone, TMAZ—thermomechanically affected zone, HAZ—heat affected zone, PM—parent material, AS—advancing side, RS—retreating side [11]. Joint (b) I_W has a visible defect in the TMAZ zone on the advancing side; this is an example of the formation of a void. The same type of defect in the microstructure also occurs in TMAZ and AS in joint (c), but its size is smaller. In other cases, the shape of the obtained nucleus is more regular for joints made using the UWFSW method. The flow of material from the nugget zone for sample (a) for the FSW method increases the probability of crack initiation during strength tests. The TMAZ zone for UWFSW connectors is more extensive, which may indicate a different temperature distribution and heat input by liquid cooling [8].

The combination of intense plastic deformation and exposure to high temperatures in the mixing zone during FSW and UWFSW leads to recrystallization and texture development in the stirred zone. Additionally, the sediment dissolves and thickens both in and around the stirred zone. However, we can observe several microstructure and precipitation phase differences between FSW and UWFSW. Starting from the lower left corner of the FSW photograph towards the opposite corner, material is characterized by a transition from a microstructure with a visible texture of plastic deformation resulting from mixing in the bonding process to a fine, dynamically recrystallized grain in the nugget zone. In the case of UWFSW, this growth does not occur to such a large extent. Additionally, the large cooling during the UWFSW rate prevents the development of new precipitates afterward. FSW images showed a microstructure typical of materials subjected to directional plastic processing. This microstructure was characterized by strongly deformed grains of the original α solution, showing a clearly elongated shape. During FSW conditions, onion ring structure was observed in the nugget zone. Particularly noteworthy is the shape of the nugget zone, which varies depending on the welding parameters and the type of method used. In the case of the two sample images considered, the differences are mainly on the retreating side of the joint of the FSW sample (c) and the UWFSW sample (d), where an arched outline of the weld nugget was observed against its irregular course in the UWFSW sample.

**Figure 5:**
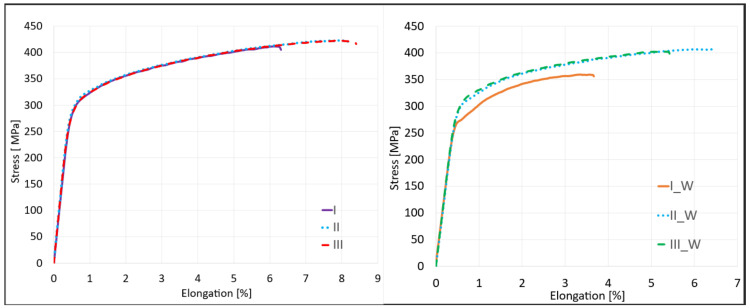
Curves obtained as a result of a static tensile test for joints obtained for the FSW and UWFSW methods.

The basic mechanical properties of joints made using the proposed set of joining parameters using the UWFSW method were determined, as well as a comparison of the results obtained for the same parameters in the FSW method. A static tensile test was performed for samples joined using the FSW and UWFSW methods (Figure 5).

Analyzing the obtained average values of tensile strength, it can be seen that in all cases for FSW, the maximum values exceeded 400 MPa. However, for UWFSW, in the case of sample I_W, the joint of which was made with parameters of 750 rpm and 200 mm/min, the average value of tensile strength was 364 MPa. In other cases, it was also higher than 400 MPa, as in FSW. The highest average strength value was recorded for joint II for FSW, amounting to 423.9 MPa. For this variant, the calculated joint efficiency was as high as 89%, and the lowest was for joint III with a value of 409.4 MPa, where the joint efficiency was 85.9%. In the UWFSW method, sample I_W differed significantly from the others in terms of the joint efficiency value, which was 76.4% compared to the remaining ones and was 8% lower. All joint efficiency values, along with the location of the cracks in the samples, are listed in Table 5.

**Figure 6:**
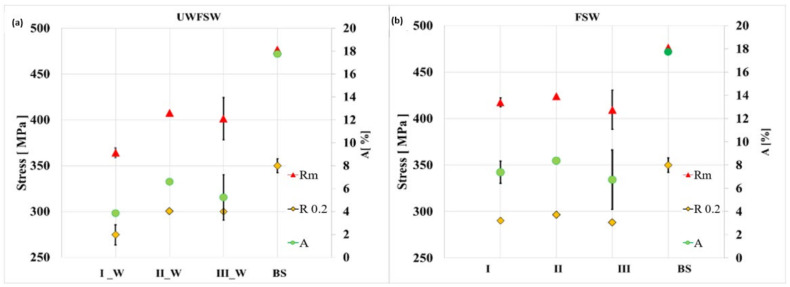
Graphs showing the obtained results of the strength properties of the AlCu4Mg1 alloy obtained during the static tensile strength for (**a**) FSW and (**b**) UWFSW.

The average values of tensile strengths, elongation at break, and yield strength, along with their standard deviations, were determined. All strength parameters obtained during the static tensile test are summarized in Table 6 and in the summary charts in Figure 6.

Additionally, the joint efficiency was also calculated as the percentage ratio of the tensile strength of the weld to the tensile strength of the basic material from Equation (2).

(2) $$EF=\frac{R_{m\;connector}}{R_{m\;mat.type}}\cdot 100\%$$

The general tendency can be associated with the amount of heat supplied in the joining process Q. In the case of samples I and I_W, the heat input coefficient was the lowest and amounted to only 3.75 (J/mm), which may indicate poor heating of the joining zone and deterioration of strength parameters. The heat input is proportional to the tool’s rotational speed and inversely proportional to the welding speed. For this reason, sets II for FSW and II_W for UWFSW obtained with a tool rotational speed of 1040 rpm and a feed speed of 143 mm/min are characterized by the most optimal heat supply of 7.27 J/mm. Samples of series III and III_W are characterized by the highest amount of heat input supplied during the welding process among the selected joints, with a value of 15 J/mm. The high rotational speed of up to 1500 rpm and the feed rate of 100 mm/min led to excessive plasticization of the material and its loss in the form of flash. The result was a reduction in the cross-section of the sample and a reduction in the strength parameters of the connection compared to sets II and II_W.

**Figure 7:**
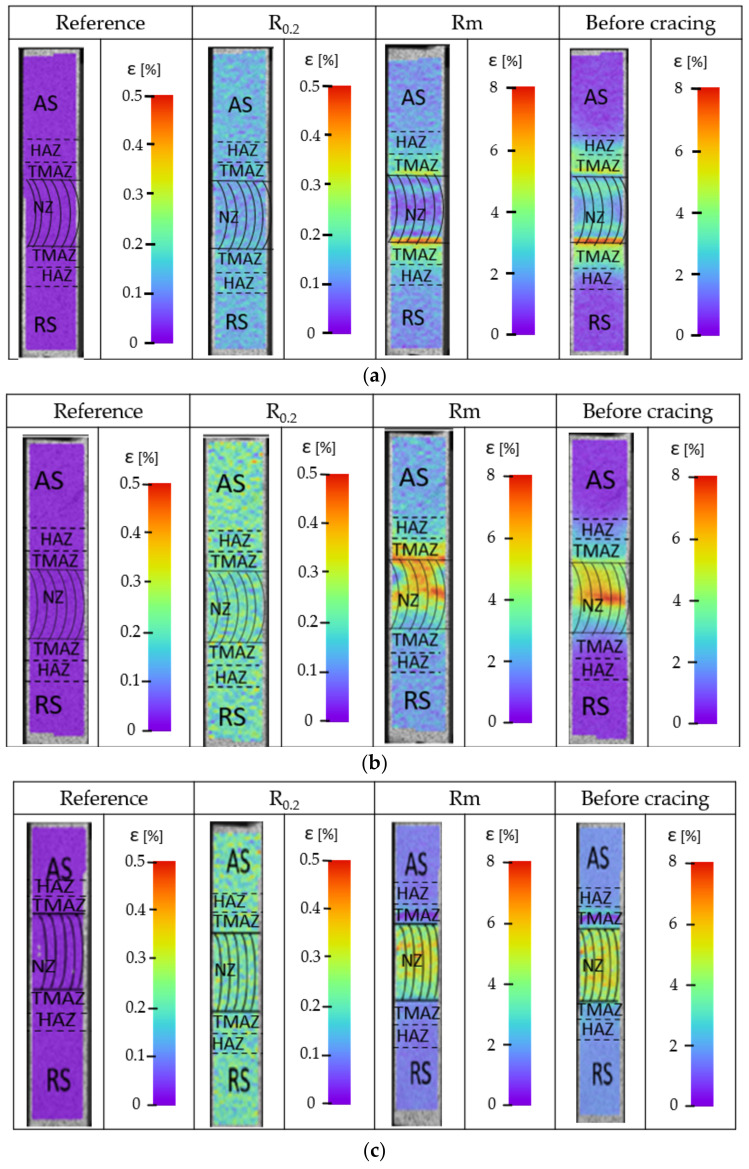

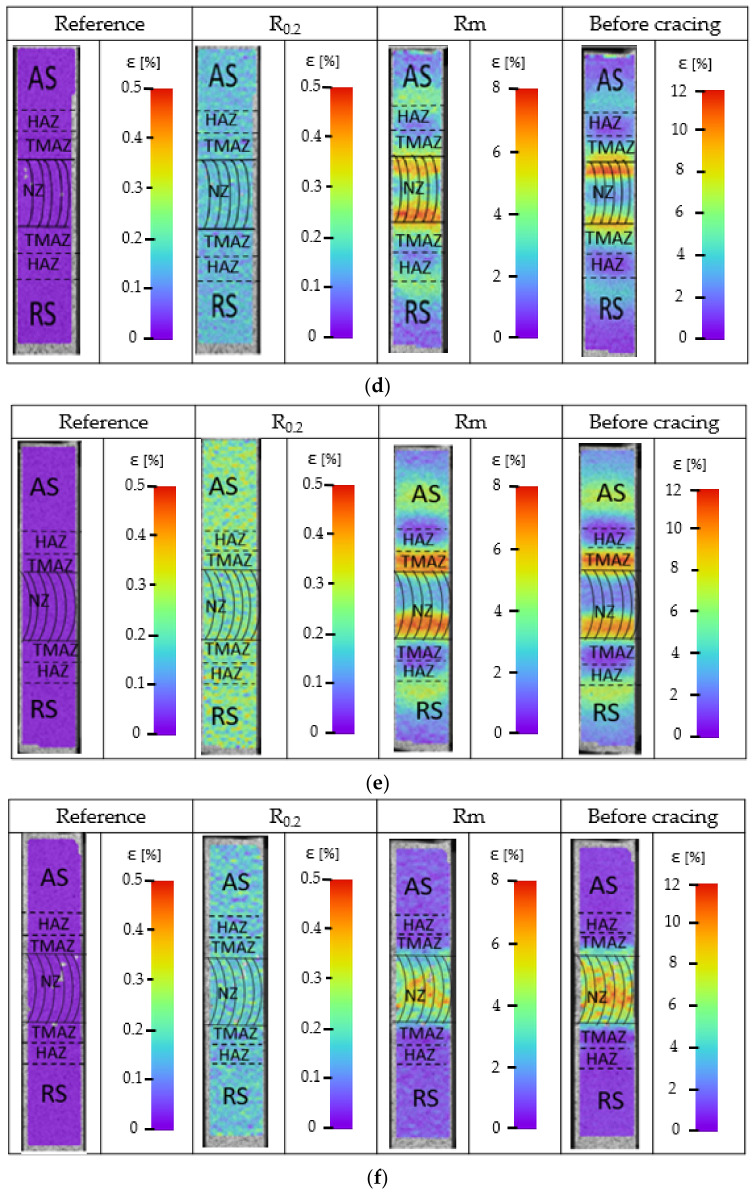
Distribution of strains recorded using digital image correlation of the tested joint from the advancing side: (**a**) I, (**b**) I_W, (**c**) II, (**d**) II_W, (**e**) III, and (**f**) III_W.

Three variants of parameters for FSW connections and, similarly, the same three sets of parameters for UWFSW connections were selected for detailed testing. Local deformation tests were carried out using digital image correlation during a static tensile test on the advancing side. The deformation results are shown in Figure 7.

The presented images were generated for four stages of the static tensile test, during elastic and plastic deformations, at maximum tensile stress, just before the sample cracked. Based on the attached photos of the samples, it is possible to trace the course of strain growth and indicate the areas with the highest strain at a given moment. Analyzing the strain distribution of the FSW and UWFSW joints of the tested alloy, several significant differences can be found. For the connection variant made using the FSW method, (a) uniform deformation is observed over the entire joint surface. Noteworthy is the band with lower deformation values located on the advancing side. This indicates the initiation of the crack in this place and is also related to the flow of material from the nugget zone indicated in the macrostructure images of the cross-sections. In the case of a joint made under water, a more even distribution of strains was obtained on both sides of the joint, with bands of the highest value visible in the TMAZ zone. Analyzing the images for variants (e) and (f), a clear band of high strain can be observed for the FSW method in the TMAZ area on the advancing side. Variant III_W has a more uniform distribution of strains in the entire NZ. The highest deformation values and stresses occur in the area of NZ. However, compared to the values recorded for FSW, they are lower by about two percent during the phase before cracking.

**Figure 8:**
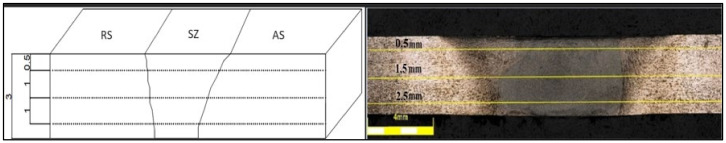
Scheme of the arrangement of hardness measurement points on the cross-section of the joint.

For the three variants of the parameters, the microhardness distribution was carried out in the samples with the FSW and UWFSW joints, along with a microscopic photo of the sample presented in Figure 8.

**Figure 9:**
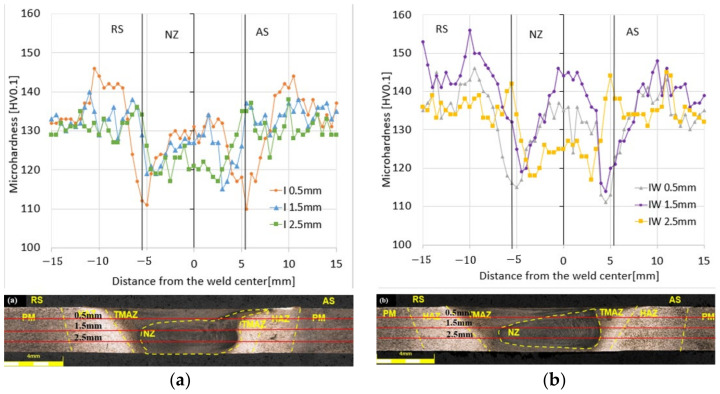

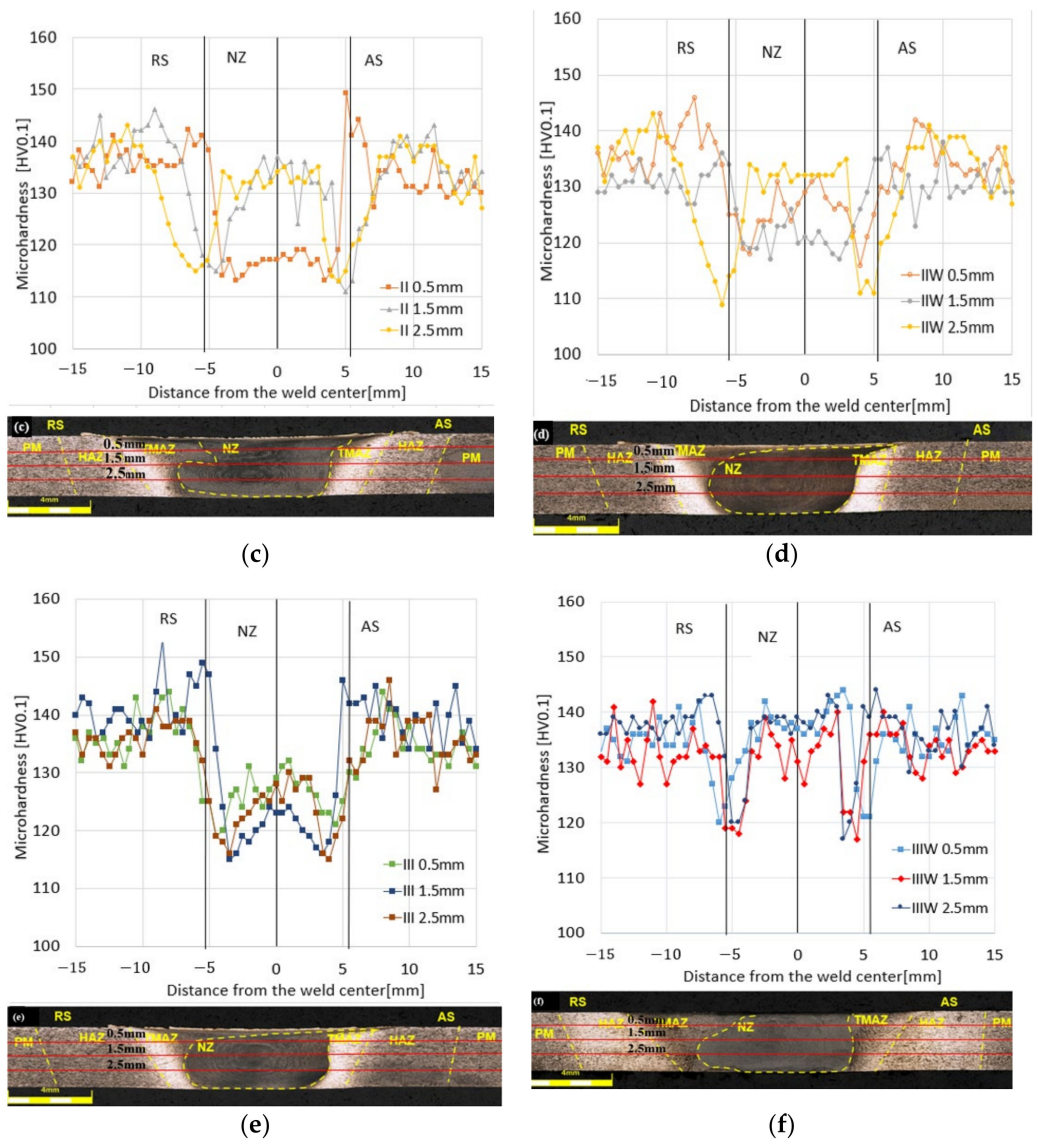
Microhardness distribution in the sample depending on the distance from the joint tip for: (**a**) I, (**b**) I_W, (**c**) II, (**d**) II_W, (**e**) III, and (**f**) III_W.

As part of the test, the following three series of impressions were made at three distances from the upper surface of the samples: 0.5 mm, 1.5 mm, and 2.5 mm (Figure 9). The determined hardness values were included in the three lines of the decomposition course. 

The following observations result from the obtained microhardness distribution charts in the tested samples. In the case of variant (a) for FSW, the value of microhardness on the RS and AS is about 135HV0.1 for distances of 1.5 mm and 2.5 mm; a much higher value is for the distance of 0.5 mm, which exceeds 140 HV0.1. The joint made with the same parameters (b) with the use of cooling has higher values of microhardness in the RS and AS areas; all are above 130 HV0.1, and for a distance of 1.5 mm, the average value in this area is about 143 HV0.1. Higher results were also obtained in the NZ zone, where the average value oscillates at about 132 HV0.1.

The microhardness of the base material for the (c) FSW method was approximately 140 ± 5 HV0.1. Similar microhardness values were also recorded for the (d) UWFSW method. As can be seen in the microhardness profile, the FSW and UWFSW processes did not reduce the microhardness of the base material in the entire joint, only in some of its areas. The nugget zone microhardness was in the range of 115–135 HV0.1 for samples from the UWFSW method. However, for the FSW method, the sample tested at a distance of 0.5 mm from the face differed significantly in terms of microhardness, where in the nugget zone values of approximately 113 HV0.1 were recorded, while for the remaining distances the values were at the level of 125–135 HV0.1. The greatest reduction in microhardness occurred at the border of the HAZ and TMAZ, which can be defined as the low hardness zone (LHZ). The microhardness value in this place is approximately 108 HV0.1 for the FSW method and is located 6 mm from the center of the joint on the downstream side of the RS. Depending on the parameters of the FSW and UWFSW processes, the LHZ zone may be located at the junction of the HAZ and the TMAZ. LHZ is the place where tensile cracks in joints occur. In the impressions made at distances of 1.5 mm and 2.5 mm from the face of the weld in the FSW method, there are slight changes in microhardness in the zone covering the core of the joint, while at a distance of 0.5 mm, the microhardness value decreases by approximately 20 HV0.1 compared to the previous ones. In the case of UWFSW, the highest microhardness value occurs at the level of 2.5 mm from the face, while the scatter of results is smaller and similar for all three distances. It can be noticed that the material closer to the edge of the tool blade is characterized by lower microhardness values. The most important conclusion is that the microhardness and location of the LHZ strongly depend on the set of joining process parameters used. The amount of heat input in the FSW and UWFSW processes is a decisive factor, as it can affect the extent of the joint zone areas and grain growth. Comparing variants III (e) and (f) III_W, much better microhardness values were obtained for the UWFSW method for both the NZ, RS, and AS areas. The value is at a similar level and is about 137 HV0.1. A decrease is seen only at the TMAZ site and is about 119 HV0.1. For the FSW method, the microhardness values in NZ are much lower and are about 125 HV0.1.

**Figure 10:**
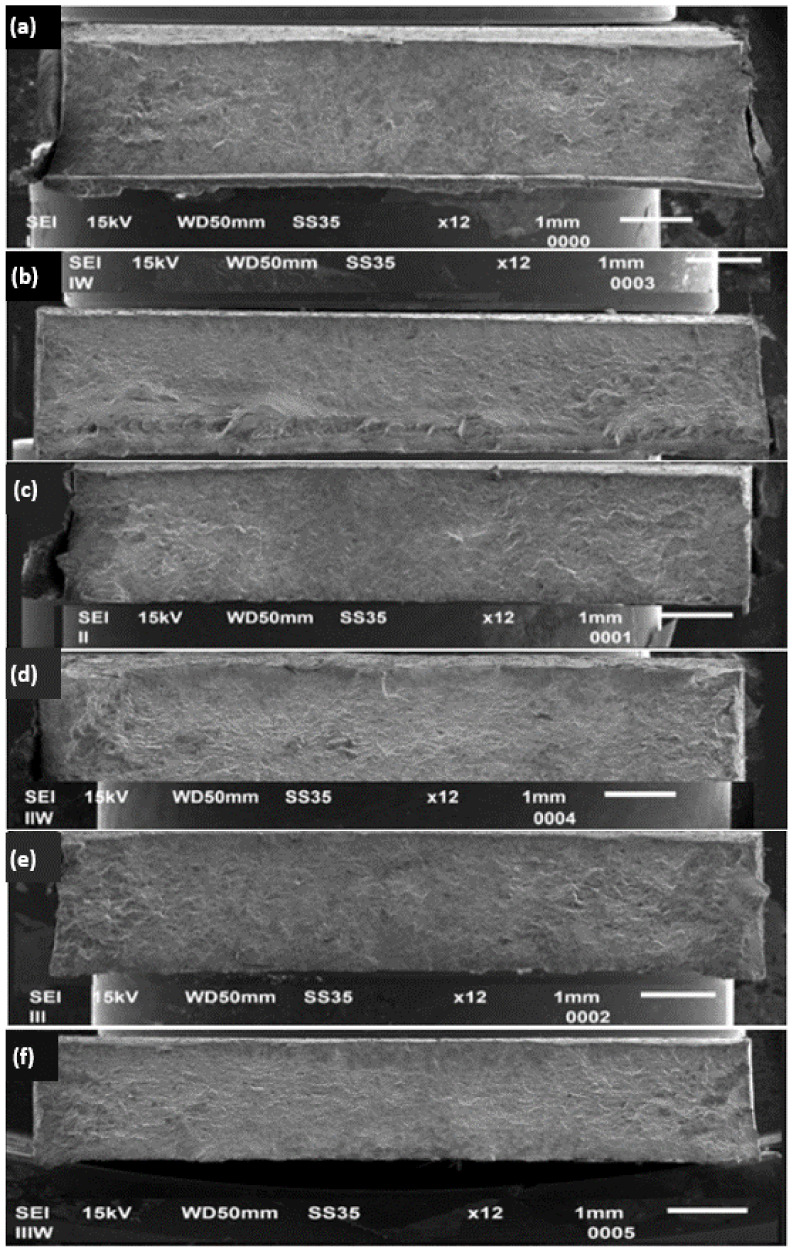
The fracture surface of the AlCu4Mg1 alloy samples, along with images of fracture surface sections of selected areas for: (**a**) I, (**b**) I_W, (**c**) II, (**d**) II_W, (**e**) III, and (**f**) III_W.

In order to assess the fracture mechanism, the sample fractures were subjected to a scanning microscopy examination. In the case of the sample I joined using the FSW method (Figure 10a), a clear source of cracking was observed in the near-surface zone. The marked crack initiation area should be associated with the place of material flow from the nugget zone, mentioned earlier during the interpretation of the macrostructure images of the cross-sections. However, in the case of the UWFSW method (Figure 10b), we can observe a typical plastic cracking mechanism. The areas on the outer parts of the samples for both methods are the so-called pits, characterized by high surface roughness, grooves, and pores. The image variants for parameters II and II_W for both FSW and UWFSW present the characteristic source of fatigue cracking, which in these cases was located at the boundary of the zones of the thermoplastic zone and the nugget zone on the advancing side. For variant (f), the crack initiation site is located in the upper right corner of the breakthrough and is characterized by an imperfection-free, smooth surface. In contrast, for the FSW variant (e), the surface is rougher, and the cracking is more banded in nature.

## 4. Conclusions

Based on the obtained test results, it can be concluded that the produced connectors are characterized by high efficiency, which exceeds 85%. The highest average strength value was recorded for joint II FSW, amounting to 423.9 MPa; for this variant, the calculated joint efficiency was as high as 89%. For the second UWFSW variant, when joined under water, a tensile strength value of 408.6 MPa and a joint efficiency of 85.6% were obtained. It was noticed that the higher the welding speed, the less the material heats up, which in extreme cases may result in incomplete mixing. Hence, the rotational speed and the linear speed are closely related and should be parameterized to compensate for each other, as in this study, when in the same variant, the rotational speed was increased while the feed was reduced. Additionally, the high welding speed in FSW results in insufficient plasticization of the material since too little heat is supplied to the process, and the use of water additionally reduces the temperature values during UWFSW. The cracking behavior of joints is random, despite repeated results in tensile tests. Therefore, research should be carried out with a larger parameter matrix. This matrix should cover the entire range of tool speed and feed. Currently, the UWFSW method allows for underwater connections but requires a broader analysis of the selection of connection parameters.

## Figures and Tables

**Table 1:** Chemical composition of the AlCu4Mg1 alloy.

Material	Chemical Composition, % Mass								
AlCu4Mg1	Si	Fe	Cu	Mn	Mg	Cr	Zn	Ti	Other
	max 0.5	max 0.5	3.8–4.9	0.3–0.9	1.2–1.8	Max 0.1	max 0.25	max 0.15	max 0.15

**Table 2:** Static strength properties of the AlCu4Mg1 alloy.

Yield Strength R_0.2_ [MPa]	Tensile Strength Rm [MPa]	Elongation at Break A [%]
331–332	459–461	17–18

**Table 4:** Dimensions of the ESAB 2902 tool used.

Schoulder profile	spiral
Schoulder diameter	16 mm
Pin profile	cylindrical with a thread
Pin length	2.7 mm
Pin diameter	5.6 mm

**Table 5:** The obtained values of the strength properties of the AlCu4Mg1 alloy obtained during the static tensile test with different sets of parameters for FSW and UWFSW.

Sample Designation	Tensile Strength Rm [MPa]	Yield Strength R_0.2_ [MPa]	Elongation at Break A [%]
BS	476.36	349.96	17.73
I	417.24	290.24	7.38
II	423.92	296.44	8.37
III	409.43	288.15	6.73
I_W	364.14	274.57	3.84
II_W	408.65	300.44	6.59
III_W	401.25	299.94	5.23

**Table 6:** Determined values of joint efficiency and crack location of tested samples for FSW and UWFSW.

Sample Designation	Joint Efficiency [%]	Crack Location
I	87.6	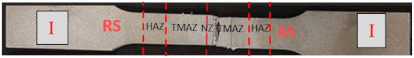
II	89	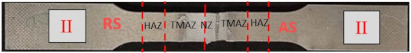
III	85.9	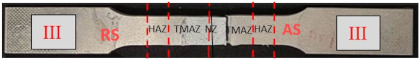
I_W	76.4	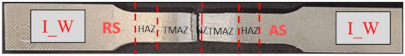
II_W	85.6	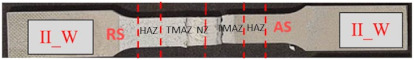
III_W	84.2	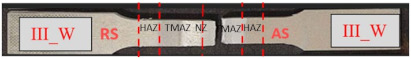

## Data Availability

Data are contained within the article.

## References

[1] Anderson K., Weritz J., Kaufman J.G. (2019). 2519: Armor Plate and Cryogenic Alloy, Properties and Selection of Aluminum Alloys.

[2] Fisher J.J., Kramer L.S., Pickens J.R. (2002). Aluminum alloy 2519 in military vehicles. Adv. Mater. Process..

[3] Lin Q., Dong W., Li Y., Zhang H., Wang Z. (2014). Microstructure Simulation of 2519 Aluminum Alloy in Multi-pass Hot Compression Process. Procedia Eng..

[4] Sobih M., Elseddig Z., Almazy K., Sallam M. (2016). Experimental Evaluation and Characterization of Electron Beam Welding of 2219 AL-Alloy. Indian J. Mater. Sci..

[5] Kashaev N., Ventzke V., Çam G. (2018). Prospects of laser beam welding and friction stir welding processes for aluminum airframe structural applications. J. Manuf. Process..

[6] Chang C.C. (2013). Microstructure in hot cracking mechanism of welded aluminium alloys. Mater. Sci. Technol..

[7] Kosturek R., Sniezek L., Grzelak K., Wachowski M. (2022). Research on the microstructure of laser beam welded Sc-modified AA2519-F extrusion. Arch. Metall. Mater..

[8] Tan Y.B., Wang X.M., Ma M., Zhang J.X., Liu W.C., Fu R.D., Xiang S. (2017). A study on microstructure and mechanical properties of AA 3003 aluminum alloy joints by underwater friction stir welding. Mater. Charact..

[9] Sidhar H., Martinez N.Y., Mishra R.S. (2016). Friction stir welding of Al–Mg–Li 1424 alloy. Mater. Des..

[10] Zhang H.J., Liu H.J., Lei Y.U. (2013). Thermal modeling of underwater friction stir welding of high strength aluminum alloy. Trans. Nonferrous Met. Soc. China.

[11] Mofd M.A., Abdollah-Zadeh A., Ghaini F.M. (2012). The efect of water cooling during dissimilar friction stir welding of Al alloy to Mg alloy. Mater. Des..

[12] Zhang H.J., Liu H.J., Yu L. (2012). Efect of water cooling on the performances of friction stir welding heat-afected zone. J. Mater. Eng. Perform..

[13] Wang B.B., Chen F.F., Liu F., Wang W.G., Xue P., Ma Z.Y. (2017). Enhanced mechanical properties of friction stir welded 5083Al-H19 joints with additional water cooling. J. Mater. Sci. Technol..

[14] Lader S.K., Baruah M., Ballav R. (2023). Significance of underwater friction stir welding on the weld integrity of thin sheets of aluminum (AA1050-O) and brass (CuZn34) joints. Mater. Sci. Eng. A.

[15] Madani T., Boukraa M., Aissani M., Chekifi T., Ziadi A., Zirari M. (2023). Experimental investigation and numerical analysis using Taguchi and ANOVA methods for underwater friction stir welding of aluminium alloy 2017 process improvement. Int. J. Press. Vessel. Pip..

[16] Datta R., Gupta S.K., Bhargava M. (2023). Comparison of underwater friction stir welded and conventional friction stir welded AA 5052 alloys based on the mechanical, formability and microstructure behaviour. Mater. Today Proc..

[17] Fratini L., Bufa G., Shivpuri R. (2010). Mechanical and metallurgical efects of in process cooling during friction stir welding of AA7075-T6 butt joints. Acta Mater..

[18] Sakurada D., Katoh K., Tokisue H. (2002). Underwater friction welding of 6061 aluminum alloy. J. Jpn. Inst. Light Met..

[19] Meikeerthy S., Ethiraj N., Neme I., Mas C. (2023). Evaluation of Pure Titanium Welded Joints Produced by Underwater Friction Stir Welding. Adv. Mater. Sci. Eng..

[20] Rathinasuriyan C., Senthil Kumar V.S. (2018). Submerged friction stir welding of 6061-T6 aluminium alloy under diferent water heads. Mater. Res..

[21] (2016). Metallic Materials-Tensile Testing.

[22] (2013). Standard Test Methods for Tension Testing of Metallic Materials.

[23] (2017). Standard Test Method for Microindentation Hardness of Materials.

